# Astrocyte Ca^2+^ Influx Negatively Regulates Neuronal Activity

**DOI:** 10.1523/ENEURO.0340-16.2017

**Published:** 2017-03-10

**Authors:** Yao V. Zhang, Kiel G. Ormerod, J. Troy Littleton

**Affiliations:** 1 The Picower Institute for Learning and Memory, Department of Brain and Cognitive Sciences, Massachusetts Institute of Technology, Cambridge, MA 02139; 2 Department of Biology, Massachusetts Institute of Technology, Cambridge, MA 02139

**Keywords:** astrocyte, Ca^2+^, *Drosophila*, GABA, GAT, Rab11

## Abstract

Maintenance of neural circuit activity requires appropriate regulation of excitatory and inhibitory synaptic transmission. Recently, glia have emerged as key partners in the modulation of neuronal excitability; however, the mechanisms by which glia regulate neuronal signaling are still being elucidated. Here, we describe an analysis of how Ca^2+^ signals within *Drosophila* astrocyte-like glia regulate excitability in the nervous system. We find that *Drosophila* astrocytes exhibit robust Ca^2+^ oscillatory activity manifested by fast, recurrent microdomain Ca^2+^ fluctuations within processes that infiltrate the synaptic neuropil. Unlike the enhanced neuronal activity and behavioral seizures that were previously observed during manipulations that trigger Ca^2+^ influx into *Drosophila* cortex glia, we find that acute induction of astrocyte Ca^2+^ influx leads to a rapid onset of behavioral paralysis and a suppression of neuronal activity. We observe that Ca^2+^ influx triggers rapid endocytosis of the GABA transporter (GAT) from astrocyte plasma membranes, suggesting that increased synaptic GABA levels contribute to the neuronal silencing and paralysis. We identify Rab11 as a novel regulator of GAT trafficking that is required for this form of activity regulation. Suppression of Rab11 function strongly offsets the reduction of neuronal activity caused by acute astrocyte Ca^2+^ influx, likely by inhibiting GAT endocytosis. Our data provide new insights into astrocyte Ca^2+^ signaling and indicate that distinct glial subtypes in the *Drosophila* brain can mediate opposing effects on neuronal excitability.

## Significance Statement

Complex brain functions require precise control of neuronal activity, which is often disrupted in neurologic disorders such as epilepsy. Here, we show that *Drosophila* astrocytes exhibit endogenous spontaneous Ca^2+^ oscillatory activity. Acute elevation of astrocyte Ca^2+^ suppresses neuronal activity and causes rapid paralysis. This is accompanied by a rapid reduction in the levels of membrane GABA transporter (GAT), a transporter for the inhibitory neurotransmitter GABA. We identify Rab11 as a novel regulator of GAT trafficking that is required for this form of Ca^2+^-dependent astrocyte regulation of neuronal activity. Our study provides new insight into the acute regulation of neuronal activity by glia.

## Introduction

The regulation of excitatory and inhibitory balance is critically important to neuronal circuit output. Mounting evidence indicates that glial cells are key regulators of neuronal activity. In particular, astrocytes have been suggested to modulate neuronal output via glia-neuron gap junctions (Nedergaard, 1994; Alvarez-Maubecin et al., 2000) or through the release of gliotransmitters, including glutamate, adenosine/ATP, and D-serine (Parpura et al., 1994; Henneberger et al., 2010; Harada et al., 2015; Ma et al., 2016). Despite being nonexcitable cells, astrocytes exhibit robust Ca^2+^ dynamics (Volterra et al., 2014; Bazargani and Attwell, 2016; Shigetomi et al., 2016). Early work in the field demonstrated that astrocytes could detect released neurotransmitters and initiate slow somatic Ca^2+^ waves secondary to Ca^2+^ release from internal stores (Cornell-Bell et al., 1990; Dani et al., 1992). However, a key role of this slower astrocytic Ca^2+^ oscillatory activity has fallen out of favor following the finding that animals lacking inositol trisphosphate receptor in astrocytes, which lack this type of glial calcium signaling, have relatively normal brain function (Petravicz et al., 2008; Agulhon et al., 2010). More recently, a form of faster, localized microdomain astrocyte Ca^2+^ oscillatory activity was discovered (Shigetomi et al., 2010). Such Ca^2+^ fluctuations are mainly contributed through influx of extracellular Ca^2+^ (Shigetomi et al., 2012; Rungta et al., 2016) and may represent a more important Ca^2+^ signaling pathway for astrocytes to regulate neural activity (Shigetomi et al., 2013).

Astrocytes also control neural activity through their function in neurotransmitter uptake. In the “tripartite synapse” model, astrocyte terminals remove neurotransmitters and terminate their action via neurotransmitter transporters (Eulenburg and Gomeza, 2010). Astrocyte uptake of GABA, the primary inhibitory neurotransmitter, via GABA transporters (GATs) is critical for proper regulation of the activity of neuronal circuits (Minelli et al., 1996). In humans, mutations in GAT that disrupt GABA uptake are associated with severe forms of epilepsy (Carvill et al., 2015). GAT has been shown to exhibit a very high turnover rate, with a third of the protein residing in a recycling pool (Wang and Quick, 2005), implying that regulation of GAT plasma membrane expression may serve as an acute mechanism to modulate neuronal activity via changes in the rate of GABA uptake. Several factors involved in GAT trafficking have been identified (Scimemi, 2014), but the mechanisms regulating fast turnover of GAT remain elusive.

*Drosophila* has several classes of glial cells (Freeman, 2015). Astrocyte-like glia (hereafter referred to as astrocytes) and cortex glia are the two main subtypes found intimately associated with neurons in the CNS (Kremer et al., 2017). It has recently been shown that *Drosophila* cortex glia exhibit near-membrane microdomain Ca^2+^ oscillatory activity (Melom and Littleton, 2013). Mutations in Zydeco, a glial-specific K^+^-dependent Na^+^/Ca^2+^ exchanger (NCKX), eliminate microdomain Ca^2+^ oscillations in cortex glia and lead to higher intracellular Ca^2+^ levels. This increase in cortex glial Ca^2+^ leads to enhanced seizure susceptibility, as does acute induction of Ca^2+^ influx through ectopic expression of TRP channels in cortex glia (Melom and Littleton, 2013). These observations have led to a model whereby increased Ca^2+^ influx into cortex glia leads to neuronal hyperexcitability. However, it is unknown whether *Drosophila* astrocytes also exhibit Ca^2+^ activity and whether astrocyte Ca^2+^ signals have a similar role in exciting neighboring neurons. In the present study, we report that *Drosophila* astrocytes exhibit spontaneous, microdomain Ca^2+^ transients, resembling those observed in their mammalian counterparts. Surprisingly, unlike cortex glia, acute Ca^2+^ influx in astrocytes causes behavioral paralysis and a rapid loss of neuronal activity. We find this suppression of neuronal activity is due in part to rapid GAT endocytosis from astrocyte membranes, leading to enhanced GABA levels in the synaptic cleft. We identify Rab11 as a key regulator of GAT trafficking downstream of astrocyte Ca^2+^ influx and find that reduction in GAT turnover via suppression of Rab11 function ameliorates the induced suppression of neuronal activity.

## Materials and Methods

### *Drosophila* stocks

All *Drosophila* stocks were maintained on standard media at room temperature (22°C). The following stocks were used: UAS-TrpA1 (BDSC 26263), Alrm-Gal4 (Doherty et al., 2009), UAS-Rab11-GFP (BDSC 8506), GAD1-Gal4 (Ng et al., 2002), UAS-Rab11-RNAi [#I, VDRC 22189 (Dietzl et al., 2007); #II, BDSC 27730 (Perkins et al., 2015)], UAS-Rab11^N124I^ (Satoh et al., 2005), repo-Gal80 (Awasaki et al., 2008), and elav-Gal80 (Yang et al., 2009). To create a stable UAS-TrpA1-myc line, UAS > stop > TrpA1-myc flies (von Philipsborn et al., 2011) were crossed with βTub85D-FLP (BDSC 7196) to excise the transcriptional stop codon. For all experiments, flies of either sex were assayed.

### Generation of transgenic flies

To construct the plasmid pBID-UASc-myrGCaMP6s, GCaMP6s cDNA (Addgene #40753) was PCR amplified by designing primers with restriction enzyme cutting sites added on each end. Plasmid pBID-UASc-myrGCaMP5G (Melom and Littleton, 2013) was digested with NotI and XbaI and ligated with GCaMP6s using standard procedure. Cloning was verified by sequence analysis (Genewiz). To generate transgenic *Drosophila*, plasmids were injected into embryos of a strain with a third chromosome attP docking site (BDSC 8622) by Bestgene.

#### Behavioral assays

For testing the temperature sensitive behavioral phenotype of adult flies, 2-d-old flies were used. A total of 10-12 flies were transferred to each vial and placed in a water bath held at the indicated temperature. The number of flies with motor impairment was observed and manually recorded for up to 5 min, with an interval of 15 s in the first minute and 30 s in the following 4 min. Paralysis of adult flies was defined by complete loss of movement. For RNAi screening conducted at 30°C, Alrm > TrpA1 flies do not show 100% penetration of the paralysis phenotype. In this case, the number of flies that showed severe motor impairment and were unable to climb up the vial wall were recorded at each time point. For testing the behavior of headless flies, heads of adult flies were severed by fine scissors under CO_2_ anesthetization. These flies were then allowed to recover at room temperature in a closed Petri dish for 1 h before being transferred to Petri dishes preheated to the indicated temperature.

#### Immunofluorescence

For immunofluorescence, 3rd instar stage larvae were dissected in HL3.1 (70 mM NaCl, 5 mM KCl, 10 mM NaHCO_3_, 4 mM MgCl_2_, 5 mM trehalose, 115 mM sucrose, and 5 mM HEPES; pH 7.2; Feng et al., 2004) and fixed for 10 min either in Bouin’s fixative or 4% paraformaldehyde. Following washes with PBST (PBS containing 0.1% Triton X-100), larval fillets were blocked for 30 min with 5% normal goat serum in PBST, then incubated with primary antibody overnight at 4°C. Fixed larvae were then washed with PBST. Secondary antibody incubation was performed at room temperature for 2 h. Samples were washed and mounted in vectashield (Vector Laboratories). Antibodies for immunostaining and their dilution were as follows: rabbit anti-GAT, 1:2000 (Stork et al., 2014); mouse anti-Repo, 1:100, (DSHB 8D12); mouse anti-Brp, 1:100 (DSHB NC82); mouse anti-GFP, 1:500 (Thermo Fisher Scientific 33-2600); DyLight 649-conjugated anti-horseradish peroxidase, 1:500 (Jackson ImmunoResearch); Alexa Fluor 488-conjugated goat anti-mouse, fluorecein isothiocyanate-conjugated goat anti-rabbit; and Alexa Fluor 546-conjugated goat anti-mouse, 1:500 (Thermo Fisher Scientific). Images were acquired on a Zeiss LSM700 Laser Scanning confocal microscope with a 40× 1.3 NA, or a 100× 1.3 NA oil-immersion objective (CarlZeiss).

#### Western blot analysis

Adult flies were snap frozen in liquid nitrogen and five heads per sample were collected and homogenized in 50 µl of 1× Laemmli loading buffer (60 mM Tris, pH 6.8; 10% glycerol; 2% SDS; 1% β-mercaptoethanol; and 0.01% bromophenol blue), followed by centrifugation at 16,000 × *g* for 10 min to clear the homogenate. Supernatant was analyzed by SDS-PAGE (Bio-Rad 456-1086) and transferred to nitrocellulose membrane. Membranes were blocked with blocking buffer (2% bovine serum albumin, 2% nonfat dry milk, 0.02% Tween 20 in TBS) and then incubated with appropriate antibodies for immunoblotting. Primary antibody incubation were performed with gentle shaking at 4°C overnight followed by four 5-min washes using TBS containing 0.01% Tween 20, secondary antibody incubation for 1 h at room temperature, and four more 5-min washes using TBS containing 0.01% Tween 20. Then the membrane was briefly rinsed with TBS, and the blot was visualized using an Odyssey infrared scanner (Li-Cor). Antibodies for Western blotting: rabbit anti-GAT, 1:5000 (gift from Marc Freeman; Stork et al., 2014); mouse anti-Tubulin, 1:60,000 (Sigma-Aldrich, clone B_5_-1-2). Alexa Fluor 680-conjugated anti-rabbit and Alexa Fluor 800-conjugated anti-mouse, 1:10,000 (Invitrogen). All antibodies were diluted using blocking buffer.

#### Electrophysiology

Extracellular recordings were obtained from dissected 3rd instar larvae using a fire-polished patch electrode. To record central pattern generator (CPG) activity, the larval brain was left intact and care was taken during dissection to avoid damaging segmental nerves. Recordings were amplified using an AxoClamp 2B amplifier and digitized via an Molecular Devices Digidata 1550 (Molecular Devices). Clampfit 6.1 software was used to record and process electrophysiological data. Larvae were constantly perfused via a gravity system with HL3.1 solution containing 1.5 mM Ca^2+^. Saline was heated using an in-line solution heater (Harvard Apparatus), controlled by a Dual Channel Bipolar Temperature Controller (CL-200A). Additionally, the recording dish was temperature controlled using a Peltier device regulated by a fan-based heat sink and a Dual Channel Temperature Controller (Koolance, Warner Instruments).

#### Live imaging of astrocyte Ca^2+^ transients

UAS-myrGCaMP6s was expressed in astrocytes using the Alrm-Gal4 driver. L2 stage larvae were mounted on their ventral side in a small drop of water on a 25 × 60 mm coverslip. A single layer of scotch tape was applied on each side of the larva as a spacer to avoid damaging the animal when an 18 × 18 mm coverslip was placed on top. An upright PerkinElmer Ultraview Vox spinning disk confocal microscope equipped with a high-speed EM CCD camera, and a 43× 1.3 NA oil objective was used for image acquisition. Velocity software was used for data analysis. When imaging, the assembly was flipped so that the ventral side of the larvae faced the objective. Time series were acquired at a speed of ∼9 Hz using a single optical plane in the neuropil layer of the larval ventral nerve cord (VNC).

#### Statistical analysis

Statistical analysis was performed using GraphPad Prism 6. Data are presented as mean ± SEM in all figures. Statistical analysis is summarized in Table 1. Superscript letters following *p* values are used to refer to the corresponding comparisons described in the table.

**Table 1:** Statistical table

	Data structure	Type of test	Observed power (α = 0.05)
a (Fig. 2A)	Non-normal distribution	Mann-Whitney test	1
b (Fig. 3A)	Normal distribution	Student’s *t* test	1
c (Fig. 3B)	Non-normal distribution	Mann-Whitney test	1
d (Fig. 3D)	Normal distribution	Student’s *t* test	0.0838271
e (Fig. 3G)	Non-normal distribution	Wilcoxon signed-rank test	0.2684
f (Fig. 4B)	Normal distribution	Student’s *t* test	0.0975257
g (Fig. 4B)	Normal distribution	Student’s *t* test	0.999942

## Results

### *Drosophila* astrocytes show microdomain Ca^2+^ transients

**Figure 1.**
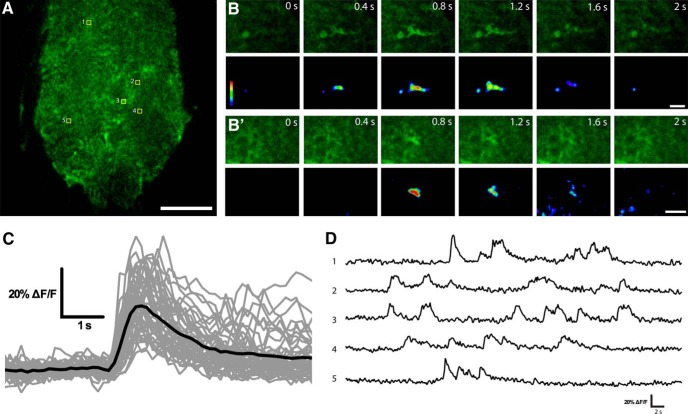
Near-membrane Ca^2+^ activity in *Drosophila* astrocytes. ***A***, Single confocal plane of larvae VNC showing the astrocyte-specific expression of myrGCaMP6s under the control of Alrm-Gal4. ***B***, ***B’***, Time-lapse image series of two microdomain astrocyte Ca^2+^ transients. ***C***, Superimposition of astrocyte Ca^2+^ traces (in gray) and their average (black line; *n* = 46 individual traces). ***D***, Sample traces of recurring Ca^2+^ transients in five different areas indicated in ***A***. Scale bars 20 µm (***A***) and 5 µm (***B*** and ***B’***).

Expression of a genetically encoded membrane targeted myristoylated Ca^2+^ indicator (GECI) in cortex glia, a type of glia that associate with neuronal cell bodies in the *Drosophila* CNS, has revealed that these cells display robust near-membrane microdomain oscillatory activities (Melom and Littleton, 2013). To determine whether astrocytes, the other major glial cell type closely associated with neurons in the *Drosophila* CNS, show similar Ca^2+^ activity, we generated transgenic lines expressing a myristoylated form of GECI, myrGCaMP6s, under the control of an astrocyte-specific promoter, Alrm-Gal4 (Fig. 1*A*; Doherty et al., 2009). We found that astrocytes display robust spontaneous Ca^2+^ oscillatory activity in membrane microdomains of processes that invaded the synaptic neuropil, largely resembling the Ca^2+^ transients observed in cortex glia (Fig. 1*B*; Movie 1). We also monitored astrocyte Ca^2+^ activity using standard dissected preparations bathed in HL3.1 saline with up to 4 mM Ca^2+^. Even when the dissections were completed in <5 min, and extreme care was taken to avoid damage to the CNS, astrocyte microdomain Ca^2+^ transients were rarely observed. Because these spontaneous astrocyte Ca^2+^ events were very sensitive to injury, we conducted Ca^2+^ imaging experiments in live, undissected L2 stage larvae that were confined between cover slips (see Materials and Methods).

Movie 1. *Drosophila* astrocyte near-membrane Ca^2+^ activity detected with myrGCaMP6s. Ca^2+^ transients were recorded from the VNC of undissected 2nd instar larvae. Video speed, 2× real time. Scale bar, 20 µm. 10.1523/ENEURO.0340-16.2017.video.1

Astrocyte near-membrane Ca^2+^ transients had an average duration of 3.69 ± 0.24 s, a half decay time of 1.00 ± 0.06 s, and a mean ΔF/F_avg_ of 34.30 ± 1.70% (*n* = 46 individual Ca^2+^ oscillatory events). These events were similar to Ca^2+^ microdomains previously described in cortex glia, which had an average duration of 1.35 s, and a mean ΔF/F_avg_ of 35% (Melom and Littleton, 2013). Recurrent Ca^2+^ transients within astrocyte processes were frequently observed in the same area (Fig. 1*D*), suggesting functional or structural subdomains where Ca^2+^ entry and removal are sequestered. Taken together, these results suggest that *Drosophila* astrocytes exhibit spontaneous Ca^2+^ oscillations that are similar to those previously described in cortex glia.

### Acute astrocyte Ca^2+^ influx via ectopic TRPA1 activation leads to paralysis

**Figure 2.**
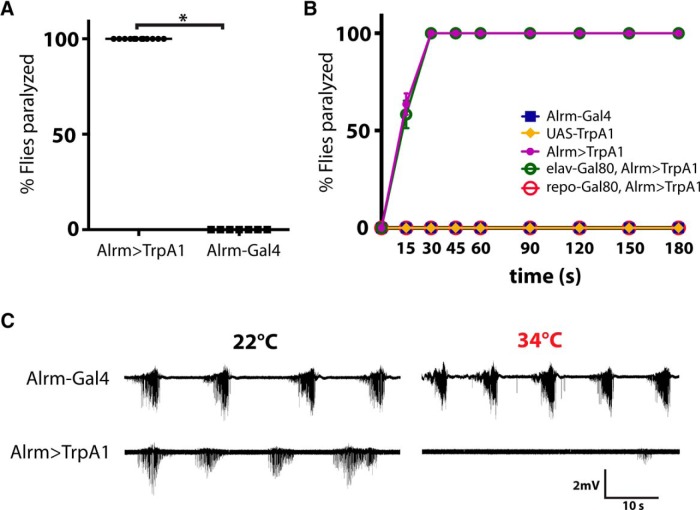
Acute astrocyte Ca^2+^ influx through TrpA1 channels suppresses neuronal activity. ***A***, All Alrm > TrpA1 flies became paralyzed within 30 s of exposure to 33°C, while control flies (Alrm-Gal4) show no behavioral defects. *n* > 80 flies per genotype; *p^a^* < 0.0001. ***B***, Time course of paralysis in adult flies of the indicated genotypes on exposure to 33°C; repo-Gal80 suppresses the paralysis in Alrm > TrpA1 background, while elav-Gal80 has no effect. *n* > 70 flies for each genotype. ***C***, Sample traces of CPG recording from larval NMJs. Postsynaptic potentials in Alrm > TrpA1 animals diminish during a temperature ramp from room temperature (22°C) to 34°C, while Alrm-Gal4 larvae maintain normal activity.

It has previously been shown that mutations in *zyd*, a *Drosophila* glial-specific K^+^-dependent Na^+^/Ca^2+^ exchanger (NCKX), led to elevated intracellular Ca^2+^ in cortex glia and enhanced seizure susceptibility, suggesting a role for glial Ca^2+^ signaling in regulating neuroexcitability (Melom and Littleton, 2013). We were interested in whether astrocyte Ca^2+^ signaling also regulates neural activity similar to that observed for cortex glia. To test this model, we used transgenic flies expressing TrpA1, a temperature-sensitive Ca^2+^ channel, under the control of a UAS promoter (UAS-TrpA1; Hamada et al., 2008). Prior studies expressing TrpA1 in cortex glia demonstrated this manipulation resulted in acute and robust seizure activity in neurons, with epileptic behavior observed in both larval and adult flies. When expressed using the astrocyte driver Alrm-Gal4, transgenic flies did not show any abnormality at room temperature (∼22°C). Strikingly, these animals became paralyzed within ∼30 s when acute Ca^2+^ influx was induced by shifting the temperature to 33°C, whereas control flies did not show any obvious behavioral defects (Fig. 2*A*; Movie 2). Similarly, acute astrocyte Ca^2+^ influx led to paralysis in 3rd instar stage Alrm > TrpA1 larvae but not in control animals (Movie 3). Pan-glial expression of Gal80, a Gal4 inhibitor, suppressed the paralysis in Alrm > TrpA1 flies, whereas pan-neuronal expression of Gal80 had no effect, indicating the observed paralysis was caused by acute Ca^2+^ influx into astrocytes rather than leaky expression in neurons (Fig. 2*B*).

Movie 2. Astrocyte Ca^2+^ influx leads to suppression of neuronal activity in adult flies. Acute paralysis was induced in Alrm > TrpA1 flies when transferred to preheated water bath held at 33°C; control flies did not show obvious behavioral deficit. Video speed is real time. 10.1523/ENEURO.0340-16.2017.video.2

Movie 3. Acute astrocyte Ca^2+^ influx leads to paralysis in *Drosophila* larvae. Third instar Alrm > TrpA1 larvae rapidly lose voluntary muscle contraction and became paralyzed following transfer to a preheated Petri dish held at 34°C. Control larvae show enhanced locomotion activity. Video speed is real time. 10.1523/ENEURO.0340-16.2017.video.3

We next recorded CPG activity from the neuromuscular junction (NMJ) of 3rd instar stage larvae. Both control and Alrm > TrpA1 animals demonstrated normal spaced bursting activity that underlies larval locomotion at room temperature (Fig. 2*C*). However, when the temperature was ramped from ∼22°C to ∼34°C, neuronal activity in the Alrm > TrpA1 animals quickly diminished, whereas controls were unaffected (Fig. 2*C*). In contrast to cortex glia, our data indicate that acute astrocyte Ca^2+^ influx suppresses neuronal activity and leads to paralysis.

### Acute astrocyte Ca^2+^ influx-induced paralysis does not require central brain function

In *Drosophila*, astrocytes extend fine processes and associate with synapses in the neuropil of both the central brain and the ventral ganglion (Muthukumar et al., 2014). The ventral ganglion is sufficient for conveying many motor functions. Headless flies with intact ventral ganglion survive for many hours and maintain posture, walk, and can learn to avoid electric shock (Booker and Quinn, 1981). It is possible that acute Ca^2+^ influx into astrocytes could suppress central brain function and cause motor impairment through descending neural circuit(s). Alternatively, the observed paralysis could be the consequence of direct interactions between astrocytes and local neurons in the ventral ganglion. To determine whether central brain function is necessary for conveying the astrocyte Ca^2+^ influx-induced paralysis, we severed the heads of adult flies and preformed behavioral tests on headless flies. Headless flies expressing TrpA1 in astrocytes rapidly became paralyzed when the temperature was shifted to 33°C (Movie 4). Therefore, acute Ca^2+^ influx in astrocytes within the ventral ganglion is sufficient to trigger paralysis; central brain function is dispensable for this process.

### A candidate screen for modifiers of astrocyte Ca^2+^ influx-induced paralysis reveals a key role for Rab11 in GAT trafficking

To understand the mechanism of acute astrocyte Ca^2+^ influx-induced suppression of neuronal activity, we conducted a candidate RNAi screen to identify modifiers of the paralysis phenotype of Alrm > TrpA1 flies. Candidate UAS-RNAi constructs were expressed in astrocytes by crossing with Alrm > TrpA1 flies, and the progeny were subjected to behavioral analysis in vials placed in a heated water bath. The screen was conducted at a lower temperature of 30°C, at which ∼80% of the Alrm > TrpA1 became paralyzed, so that both suppressors and enhancers of motor impairment could be identified. We assembled a collection of ∼140 RNAi lines for proteins that are (1) involved in vesicle transport, (2) enriched in astrocytes, or (3) involved in regulation of Ca^2+^ signaling. Using this collection, we identified 13 RNAi lines that either enhanced or suppressed the motor impairments of Alrm > TrpA1 flies (Table 2).

**Table 2:** A screen for modifiers of the TS paralysis phenotype of Alrm > TrpA1 flies

Gene product	CG no.	Effect	Function	RNAi stock no.
Esyt2	CG6643	Enhance	ER-PM tethering	v28418, v28419
SCAMP	CG9195	Enhance	SV trafficking	v9130
Nep4	CG4058	Enhance	Metalloendopeptidase	v16669, v100189
Syt4	CG10047	Suppress	Vesicle trafficking	v33317, BL26730
Syx1A	CG31136	Suppress	SNARE; Vesicle trafficking	v33113
Syt12	CG10617	Suppress	Vesicle trafficking	v47503
α-SNAP	CG6625	Suppress	SNARE disassembly	v101341, BL29587
Rab11	CG5771	Suppress	Endosome trafficking	v22198, BL27730

**Figure 3.**
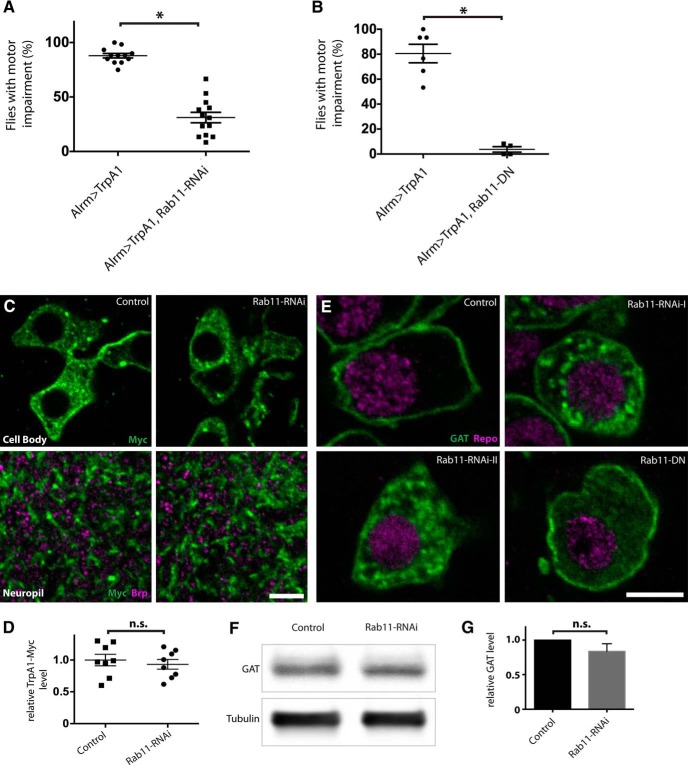
Suppression of Rab11 function ameliorates astrocyte Ca^2+^ influx-induced behavioral defects and impairs GAT trafficking. ***A***, Quantification of paralyzed flies when exposed to 30°C, showing *rab11-*knockdown suppressed the paralysis in Alrm > TrpA1 flies. *n* > 100 flies per genotype; *p^b^* < 0.00001. ***B***, Quantification of paralyzed flies when Alrm > TrpA1 and Alrm > TrpA1, Rab11-DN flies were transferred to 30°C. *n* > 45 flies per genotype; *p^c^* = 0.0095. ***C***, Single optical section showing astrocyte cell body and neuropil in the VNCs of Alrm > TrpA1-myc (control) and Alrm > TrpA1-myc, Rab11-RNAi (Rab11-RNAi) larvae stained with antibodies against Myc (green) and Brp (magenta). Note the apposition between synapses and astrocyte processes in the neuropil. ***D***, Quantification of relative TrpA1-Myc level, normalized to control. *n* = 8 individual optical planes for each groups; *p* ^d^ = 0.5761. ***E***, Optical sections showing GAT localization in astrocytes of larval VNCs stained with antibodies against GAT (green) and Repo (magenta) in control and when Rab11-RNAi or Rab11-DN is expressed in astrocytes. ***F***, Western blotting of adult heads showing the effect of Rab11-RNAi expression in astrocytes on GAT level. ***G***, Quantification of ***F***. *n* = 6 experiments; *p* ^e^ = 0.3125. Scale bars in ***C*** and ***E***, 5 µm.

Among the RNAi lines that suppressed the paralysis phenotype of Alrm > TrpA1 flies, Rab11-knockdown displayed the strongest effect (Fig. 3*A*; Alrm > TrpA1: 87.92 ± 2.043%; Alrm > TrpA1, Rab11-RNAi: 31.12 ± 4.813%; *n* > 100 flies for both groups; *p* ^b^ < 0.0001). We verified the RNAi knockdown result using a dominant-negative mutant form of Rab11, Rab11^N124I^ (Satoh et al., 2005). Overexpression of UAS-Rab11^N124I^ in astrocytes resulted in a comparable suppression of the paralysis in Alrm > TrpA1 flies (Fig. 3*B*; Alrm > TrpA1: 80.56 ± 7.425%; Alrm > TrpA1, Rab11-DN: 3.75 ± 2.192%; *n* > 45 flies for both groups; *p* ^c^ = 0.0095). Rab11 is a member of the Rab family of small-GTPases, and it is crucial for recycling endosome function and post-Golgi transport (Welz et al., 2014). It is possible that the reduced susceptibility to paralysis after suppression of Rab11 function in astrocytes is a result of impaired TrpA1 transport to astrocyte membranes and hence reduced Ca^2+^ influx on temperature shifts. However, following expression of a UAS-TrpA1-myc transgene, we did not observe alteration in TrpA1-Myc localization (Fig. 3*C*) or intensity with *rab11* knockdown (Fig. 3*D*; control: 1 ± 0.0903, Rab11-RNAi: 0.9323 ± 0.0764, *n* = 8 single optical sections for each group, *p* ^d^ = 0.5761).

One of the major functions of astrocytes is to uptake neurotransmitters following synaptic activity (Eulenburg and Gomeza, 2010). *Drosophila* astrocytes express GAT, the transporter responsible for synaptic clearance of the major inhibitory neurotransmitter GABA (Stork et al., 2014). It has been shown that membrane GAT levels are highly dynamic, and changes in GAT level can regulate neuronal excitability (Wang and Quick, 2005; Muthukumar et al., 2014). If GAT function or surface levels were suppressed by Ca^2+^ influx into astrocytes, one might predict an acute behavioral paralysis due to high synaptic GABA levels and a general suppression of neuronal excitability. We tested whether suppression of Rab11 alters the transport or localization of GAT. Indeed, Rab11-knockdown in astrocytes led to accumulation of large amounts of GAT-containing vesicles in the cytoplasm, although GAT is normally predominantly localized to the plasma membrane (Fig. 3*E*). Overexpression of Rab11^N124I^ caused a similar defect in GAT localization (Fig. 3*E*). We also assayed whether suppression of Rab11 function altered the overall level of GAT. Compared with controls, *rab11*-knockdown animals have similar levels of overall GAT (Fig. 3*F*,*G*; control: 1, Rab11-RNAi: 0.8368 ± 0.1102, *n* = 6 experiments, *p* ^e^ = 0.3125). These data suggest that Rab11 is a novel regulator of GAT trafficking and likely ameliorates the acute astrocyte Ca^2+^ influx-induced paralysis of Alrm > TrpA1 flies by indirectly regulating GABA uptake.

### Acute astrocyte Ca^2+^ influx leads to reduced membrane GAT

**Figure 4.**
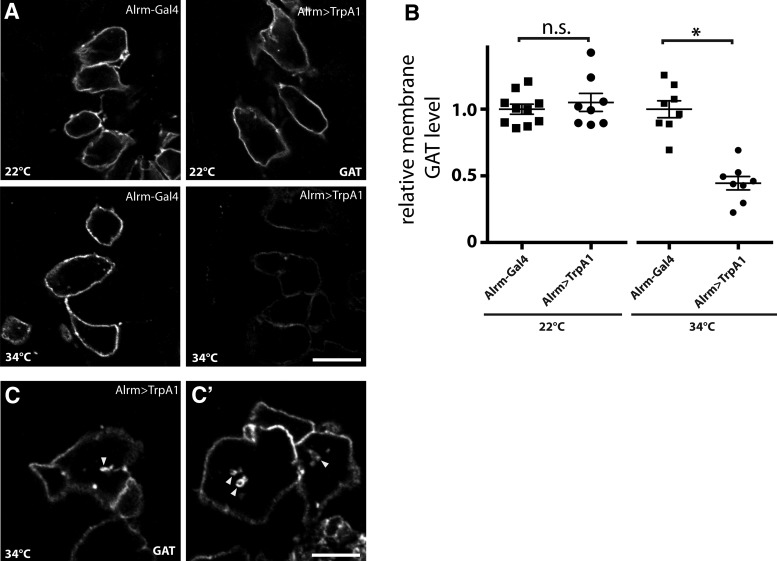
Astrocyte Ca^2+^ influx leads to reduced membrane GAT. ***A***, Confocal images of larval VNC showing astrocyte membrane GAT level in control and Alrm > TrpA1 animals at room temperature (22°C) or 34°C. ***B***, Quantification of relative GAT level shown in ***A***, normalized to Alrm-Gal4 animals at each temperature. *n* = 8-10 individual optical planes for all groups; *p* ^f^ = 0.4974 and *p* ^g^ < 0.0001, respectively. ***C***, ***C’***, Confocal images with longer exposure showing GAT-containing vesicles (arrowheads) forming in the cytoplasm of Alrm > TrpA1 larvae exposed to 34°C. Scale bars in **A** and **C**, 5 µm.

At room temperature, Alrm > TrpA1 larvae display normal GAT expression and localization to astrocyte plasma membrane processes (Fig. 4*A*,*B*; Alrm-Gal4: 1 ± 0.03759, Alrm > TrpA1: 1.051 ± 0.06748, *n* = 8-10 single optical sections for each group, *p* ^f^ = 0.4974). However, after subjecting animals to 33°C, Alrm > TrpA1 larvae showed a severe reduction of surface GAT expression compared with controls (Fig. 4*A*,*B*; Alrm-Gal4: 1 ± 0.06333, Alrm > TrpA1: 0.4451 ± 0.05023, *n* = 8 single optical sections for each group, *p* ^g^ < 0.0001). Large, GAT-containing membranous structures were frequently observed in the cytoplasm following acute Ca^2+^ influx in astrocytes, indicating Ca^2+^ elevation may drive GAT endocytosis (Fig. 4*C*).

**Figure 5.**
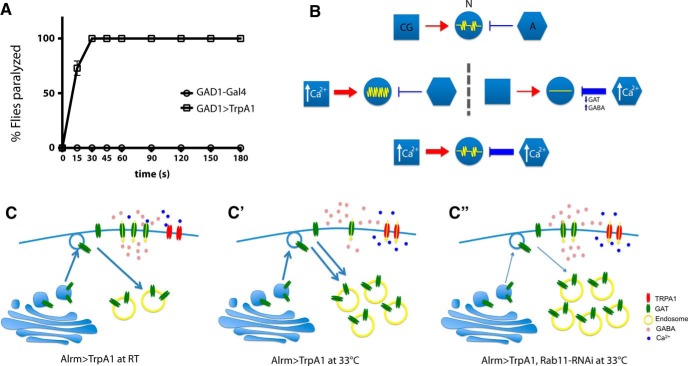
Excessive GABA leads to suppression of neuronal activity. ***A***, Quantification of paralyzed flies on exposure to 30°C. Ectopic activation of GABAergic neurons via GAD1-Gal4-induced expression of TrpA1 leads to acute suppression of neuronal activity and paralysis. ***B***, Distinct impact of cortex glia and astrocyte Ca^2+^ signals on neuronal activity. Ca^2+^ influx in cortex glia leads to increased neural activity and seizure-like behavior, whereas enhanced astrocyte Ca^2+^ signal causes suppression of neuronal activity and paralysis. Together, they constitute a Ca^2+^-dependent glial mechanism to fine-tune neuronal function. ***C-C”***, Model of how astrocyte Ca^2+^ signaling regulates neuronal activity. Astrocyte Ca^2+^ influx leads to acute endocytosis of membrane GAT, reduced GABA uptake, and suppression of neuronal activity. Inhibition of Rab11 function reduces the removal of membrane GAT and sustains GABA uptake, hence ameliorating the paralysis caused by astrocyte Ca^2+^ influx.

These results suggest a Ca^2+^-dependent endocytotic pathway dynamically regulates membrane GAT levels. We hypothesize that excessive synaptic GABA due to the reduction in astrocyte surface GAT levels triggers the Ca^2+^ influx-induced paralysis. To test whether increased GABAergic signaling could lead to rapid paralysis, we expressed TrpA1 using a GABAergic neuron driver, GAD1-Gal4 (Ng et al., 2002). These flies became paralyzed within ∼30 s after being transferred to 30°C, indicating excessive GABA synaptic levels result in a strong suppression of neuronal activity and rapid behavioral paralysis in *Drosophila* (Fig. 5*A*).

## Discussion

In the present study, we investigated Ca^2+^ influx in *Drosophila* astrocytes and the role of astrocyte Ca^2+^ signals in regulating neuronal activity. We found that similar to cortex glia (Melom and Littleton, 2013), *Drosophila* astrocytes exhibit spontaneous Ca^2+^ transients. However, in contrast to cortex glia, acute astrocyte Ca^2+^ influx leads to suppression of neuronal activity. Elevated cytoplasmic Ca^2+^ in astrocytes is associated with fast endocytosis of GAT and a reduction in surface expression, which is likely to disrupt GABA uptake and induce rapid paralysis. Suppression of Rab11 led to a disruption in GAT distribution and ameliorated the paralysis caused by astrocyte Ca^2+^ influx, revealing a novel mechanism of Rab11-dependent control of membrane GAT and neuronal activity.

### *Drosophila* astrocytes demonstrate spontaneous microdomain Ca^2+^ activity

Current data indicate that astrocytes display diverse forms of intracellular Ca^2+^ oscillations through distinct cellular mechanisms (Bazargani and Attwell, 2016). Changes in astrocyte Ca^2+^ levels could be a response to neuronal activity. In isolated rat retinas, light flashing causes an increase in Ca^2+^ in retinal glial cells (Newman, 2005). In the cortex of anesthetized mice, astrocytes were also found to elevate intracellular Ca^2+^ in response to whisker stimulation (Wang et al., 2006). However, the Ca^2+^ oscillatory activities that we observe in *Drosophila* astrocytes appear to more closely resemble another form of fast, microdomain Ca^2+^ fluctuation, which has not yet been demonstrated to correlate with neuronal activity. In a rodent neuron-astrocyte coculture system, Ca^2+^ oscillations in astrocytes appeared to be mediated by spontaneous opening of TRPA1 channels independent of neuronal spiking (Shigetomi et al., 2012). Another study using acute mouse hippocampal brain slices also showed that pharmacological blockage of neuronal activity did not affect either the frequency or the amplitude of localized Ca^2+^ transients at fine processes of CA1 astrocytes (Rungta et al., 2016).

It is interesting that Ca^2+^ oscillations in *Drosophila* astrocytes bear remarkable similarity with those observed in cortex glia (Melom and Littleton, 2013), despite the distinction in the morphology between these two types of glial cells. The similar patterns of Ca^2+^ oscillations may suggest a common cellular mechanism for their origin. Unlike astrocytes, cortex glia cells are segregated from the synaptic neuropil and, instead, enwrap neuronal cell bodies in the cortex region of the CNS with their thin processes (Ito et al., 1995). It is unclear whether cortex glial might directly sense neuronal activity as a trigger for Ca^2+^ oscillations due to their lack of direct contact with chemical synapses. With the caveat that we could not use pharmacological methods to directly test the origin of astrocyte Ca^2+^ transients due to their sensitivity to injury, these microdomain Ca^2+^ fluctuations are likely a result of spontaneous glial channel opening rather than being directly triggered by neuronal activity.

### Distinct functions of glial Ca^2+^ signals on neuronal activity

We find that acute Ca^2+^ influx into astrocytes leads to suppression of neuronal activity, an effect opposite to the enhancement of neuronal firing following elevated cortex glia Ca^2+^ signaling (Melom and Littleton, 2013). Although the exact mechanism is unclear, increased cortex glial Ca^2+^ level may trigger excessive neuronal activity through their ability to influence the environment around neuronal cell bodies, or through gliotransmission that activates surface receptors on neuronal cell bodies. Based on our results, astrocyte Ca^2+^ influx appears to balance cortex glia excitation through inhibition of neuronal activity, and together they constitute a glial mechanism to fine-tune the balance between excitatory and inhibitory signals to maintain normal neuronal excitability (Fig. 5*B*).

Perturbations in glial Ca^2+^ activity have been implicated in several forms of neurologic disorders (Nedergaard et al., 2010; Shigetomi et al., 2016). For example, it was found that both the basal astrocyte Ca^2+^ level and the frequency of somatic Ca^2+^ activity increase in a mouse model of Alzheimer’s disease, which might contribute to alterations in brain function (Kuchibhotla et al., 2009). Although we did not find evidence that the transient induction of astrocyte Ca^2+^ influx used in our study resulted in long-lasting pathology (data not shown), it will be interesting to explore the role of chronic increases in glial Ca^2+^ signals in the pathogenesis of neurologic diseases using the *Drosophila* model.

In the *Drosophila* CNS, astrocytes invade the entire neuropil with their meshwork of fine processes, and each astrocyte establishes unique and stereotypical territory (Muthukumar et al., 2014; Stork et al., 2014). Although much is known about the distinct origin and functions of astrocytes in different mammalian brain regions (Bayraktar et al., 2014), the functional heterogeneity and/or redundancy of astrocytes in *Drosophila* is still largely unexplored. Here, as a first step, we show that astrocytes in the ventral ganglion are sufficient to convey the suppression of neuronal activity caused by acute Ca^2+^ influx.

### A mechanism regulating astrocyte membrane GAT and neuroactivity

Modulation of GABA uptake is an important glial mechanism to actively participate in the regulation of neuronal activity. It has been previously shown that global GAT levels in *Drosophila* astrocytes are developmentally regulated and sensitive to GABAergic neuronal output during synaptogenesis (Muthukumar et al., 2014). Suppression of metabotropic GABA_B_ receptor signaling reduces GAT levels and suppresses seizure induction in hyperexcitable *Drosophila* seizure mutants, likely through dampening the rate of GABA uptake (Muthukumar et al., 2014).

Our study suggests the existence of a new mechanism for astrocytes to acutely regulate neuronal activity through fast modulation of GAT turnover. Compared with other known membrane proteins, GAT has one of the highest turnover rates (Tanner and Lienhard, 1987; Harwood and Pellarin, 1997; Menne et al., 2002; Wang and Quick, 2005). Plasma membrane GAT levels are dynamically regulated via a balance between fast endocytosis and exocytosis in primary rat cortical neuron cultures (Wang and Quick, 2005). Pharmacological activation of protein kinase C (PKC) can trigger enhanced endocytosis of GAT, leading to reduced membrane expression and reduction in GABA uptake (Wang and Quick, 2005). Here, our results suggest that astrocyte Ca^2+^ influx induces a rapid decrease in membrane GAT levels via endocytosis, which correlates with acute suppression of neural activity, representing a mechanism for glial cells to actively modulate neuronal function.

Our data reveal Rab11 as a novel regulator of GAT trafficking in *Drosophila* astrocytes. The accumulation of GAT-containing vesicles in *rab11*-knowdown animals likely represents a strong suppression of post-Golgi transport of GAT to the plasma membrane. However, it is important to note that suppression of Rab11 function did not totally abolish GAT localization to astrocyte membranes (Fig. 3*D*). Consistently, *rab11*-knowdown flies do not have strong locomotion defects like GAT-deficient animals (Stork et al., 2014). Indeed, it has been shown that GAT is delivered to the plasma membrane via multiple pathways (Reiterer et al., 2008). Since *rab11*-knockdown did not alter total GAT levels (Fig. 3*E*,*F*), constitutive membrane GAT in these animals seems sufficient to support baseline GABA uptake. Although primarily considered a regulator of recycling endosomes, Rab11 has been shown to promote endocytosis through indirect mechanisms. In the *Drosophila* eye, suppression of Rab11 function decreases the rate of endocytosis and eliminates the formation of endocytosis-dependent multivesicular bodies (Satoh et al., 2005). Ectopic expression of a dominant negative form of Rab11 also disrupts endocytosis in cultured *Drosophila* Garland cells (Satoh et al., 2005). Consistent with these observations, our data support a model in which suppression of Rab11 function blocks GAT endocytosis triggered by astrocyte Ca^2+^ influx, leading to sustained GABA uptake and a suppression of the paralysis and loss of neural activity normally observed following excessive astrocyte Ca^2+^ influx (Fig. 5*C*).

Taken together, our study reveals an important function of glial Ca^2+^ signaling in regulating neuronal activity. Acute modulation of the rate of neurotransmitter uptake through Ca^2+^-dependent glial signaling provides a robust mechanism for astrocytes to actively modulate the function of neural circuits.

Movie 4. Ca^2+^ influx in adult ventral ganglion astrocytes causes paralysis. Headless adult Alrm > TrpA1 flies show paralysis when transfer to a preheated Petri dish held at 33°C. The video started 30 s after the flies were transferred to the Petri dish. Video speed is real time. 10.1523/ENEURO.0340-16.2017.video.4

## Synthesis

Reviewing Editor: Maiken Nedergaard, University of Rochester Medical Center Decisions are customarily a result of the Reviewing Editor and the peer reviewers coming together and discussing their recommendations until a consensus is reached. When revisions are invited, a fact-based synthesis statement explaining their decision and outlining what is needed to prepare a revision will be listed below. The following reviewer(s) agreed to reveal their identity: Leonard Khiroug

Synthesis of Reviews:

Your manuscript was well-received by both reviewers. The reviewers have raised several important questions that will need to be addressed. In addition, I will ask for a comparison of spontaneous and Ca2+ increases in transgenic flies expressing TrpA1 under the control of the temperature sensitive promoter. I worry that the calcium increases are pathological high and/or long-lasting and it will important to also address this point in the discussion.

Reviewer #1 Comments:

The article under review is well-written and advances the field of glial-neuronal communication in at least two ways: first, it provides a nice example of network fine-tuning via opposite effects of two sub-populations of glial cells (cortical glia and astrocyte-like glia) on neuronal activity; second, it provides novel mechanistic details on the link between astrocytic Ca2+ signaling and neuronal network silencing (via Rab11-mediated internalization of GAT resulting in increased extracellular GABA and neuronal inhibition).

There are no major concerns.

Minor concerns and suggestions are listed below:

1) Regarding the power analysis for the statistics shown in Fig.3F: is the group size (n=6) and the alpha value (0.2684, Table 1) sufficient to justify the conclusion that the overall GAT level is NOT changed by Rab11-RNAi? Please elaborate.

2) The use of the term "astrocyte-like glia" seems more appropriate than "astrocytes", however it is also too long for repeated use in the text; to address this, I suggest adding a sentence like "...Drosophila astrocyte-like glia (also referred to as "astrocytes" throughout this paper), ..."

3) In line 107, please add details on how exactly the behavior was recorded (was it a manual count/score? any automation of behavior analysis?)

4) In line 187, please explain in which manner did injury affect the spontaneous Ca2+ events (e.g., were they more frequent upon injury? did the time course of individual events change? etc)

5) While in general the article is very well written, I would like to suggest a couple of changes: first, on a number of occasions (e.g., lines 212, 240, 252, 304 and 544) the word "paralyze" is used in active voice, which does not make sense because flies "get paralyzed" or "become paralyzed" rather than "paralyze someone"; second, there are some mismatches between plural versus singular nouns (e.g., lines 4, 21, 64, etc).

6) Please add some quantification of TrpA1 membrane localization (similar to Fig.4B), to support the qualitative evidence shown in Fig.3C and the conclusion about lack of TrpA1 translocation.

7) I would recommend editing movies 2, 3 and 4 by adding a mark that indicates the start (and duration) of the temperature ramp from RT to 30-34C.

Reviewer #2 Comments:

In the paper entitled "Astrocyte Ca2+ Influx Negatively Regulates Neuronal Activity", Authors are suggesting that Ca influx in Drosophila astrocytes negatively regulates neural activity via GAT internalization through Rab11, caused transient paralysis.

The purpose of this study is potentially important in this research field. The manuscript is well written enough, however some of the points need to be addressed.

Suggestions are details as follows;

Major points

1 Authors mentioned astrocytic Ca2+ elevation trigger GAT internalization. I wonder the spatial distribution between Ca2+ hot spot and GABAergic terminal. In Fig3 and 4 authors showed that GAT internalization occurred through the whole cell. Does it happen through the down regulation of Rab11 activity in 30 sec? What is the mechanism that induced Rab11 inactivation through the local Ca2+ elevation.

2 If so, how does local Ca2+ elevation trigger the down regulation of Rab11

3 GAT expression in Fig3 and 4 seemed to be different. Fig 3 with Rab11 inhibition seemed to have GAT expression in cell soma but seem to have weak staining or less expression in Fig4. Author should mention for the reason.

4 Author should quantitatively measure the membrane expression of GAT or expression of Rab11 with the activation of astrocyte.

5 If possible, author have better to take correlation between Ca2+ level and GAT internalization. For instance, see if they have any correlation with the frequency of Ca2+ transients and GAT staining at membrane.

Minor points

1. Authors lack the Figure number

2. In Fig1, author should put the outline of astrocyte to make recognize cell contour.

3. Authors have better to compare the Ca2+ in Alrm-Gal4 transgenic Drosophila at different temperature to confirm their activation.

4. Author should show better staining for neuropils in Fig3 to observe their co-localization with astrocyte.

5. Misspelling "myrisotoylated (177)"

6. Misspelling "TrapA1 (line252)"

7. Misspelling "RNA11-RNAi (Fig5.C")"
